# Epidemiologic Aspects of Animal Bite, Rabies, and Predictors of Delay in Post-exposure Prophylaxis: A National Registry-based Study in Iran

**DOI:** 10.34172/jrhs.2023.118

**Published:** 2023-06-29

**Authors:** Salman Khazaei, Mohammad Reza Shirzadi, Behzad Amiri, Jamshid Pourmozafari, Erfan Ayubi

**Affiliations:** ^1^Department of Epidemiology, School of Public Health, Hamadan University of Medical Sciences, Hamadan, Iran; ^2^Research Center for Health Sciences, Hamadan University of Medical Sciences, Hamadan, Iran; ^3^Center for Communicable Diseases Management, Ministry of Health and Medical Education, Tehran, Iran; ^4^Social Determinants of Health Research Center, Hamadan University of Medical Sciences, Hamadan, Iran

**Keywords:** Rabies, Post-exposure prophylaxis, Epidemiologic factors, Iran

## Abstract

**Background:** The increasing trend in animal bites and rabies in recent years makes the disease a public health concern in Iran. The objectives of the current study were to investigate the epidemiologic aspects of the animal bite and determine the associated risk factors of the delay in initiation of post-exposure prophylaxis (PEP) against rabies in Iran.

**Study Design:** National registry-based cross-sectional study.

**Methods:** This study included all registered cases of animal bites between March 2021 and March 2022 at the Ministry of Health and Medical Education in Iran. We retrieved epidemiologic data on person, time, place, and PEP outcome.

**Results:** A total of 260470 animal bite cases (approximately 334 per 100000 populations, and 11 deaths) were registered during the study period. About 77.2% of them were reported in males, 4.3% in children aged less than 5 years, 56.4% occurred in urban areas, 98% in domestic animals, and mostly in north and northeast areas of Iran. Additionally, 2.8% of cases had a delay of more than 48 hours in the initiation of PEP. Significant determinants of the increase in delay were female gender (OR=1.43, 95% CI: 1.36, 1.51, *P*<0.001), foreign nationality (OR=1.19, 95% CI: 1.01, 1.12, *P*=0.001), rural residence (OR=1.07, 95% CI: 1.01, 1.12, *P*=0.010), and the wild animals (OR=1.22, 95% CI: 1.12, 1.34, *P*<0.001).

**Conclusion:** The observed frequency of animal bites in a year indicates a serious public health concern and the need for targeted interventions, especially in at-risk areas and vulnerable populations.

## Background

 Rabies is one of the most common zoonotic diseases, which is caused by the *Lyssaviru*s genus of the *Rhabdoviridae* family.^1^ This virus affects wild and domesticated mammals, and although this disease is vaccine preventable, it is estimated that about 15 000 human deaths from rabies occur worldwide every year.^2^ The burden of rabies is closely associated with the sociodemographic level. Southeast Asia, Africa, and Eastern Europe have the highest rate of rabies, in which the disease is endemic in domestic dog populations.^2^ In addition to the lethality of the disease in humans, the occurrence of disease in livestock also causes significant economic burden.^3^ In the world, 3.7 million disability-adjusted life years are attributed to rabies every year.^4^

 Studies conducted in different parts of the country show that the incidence of animal bites is increasing in recent years. The results of a meta-analysis study on 34 national studies showed that the estimated incidence of animal bites in the country is 13.2 per 1000 population, and it is three times higher in men than in women. In this study, the rate of animal bite in rural areas was 17.45 compared to 4.35 people per 1000 population in urban areas. The number of animal bites was higher in domestic animals and the highest number of bites belonged to dogs.^5^ Rabies in Iran has complex transmission dynamics through dogs and wildlife population and is also common in domestic animals. Rabies virus circulates in almost all 31 provinces of the country.^6^ However, the country’s plan for surveillance and control of rabies in humans has major achievements such as mapping the geographical distribution, determining the provinces with the higher incidence of animal bites, and decreasing the number of rabies-related deaths.^7^

 By proper vaccination even after exposure, this disease can be prevented. Therefore, in countries where rabies is endemic such as Iran, enhancement of the vaccination schedule is essential. Factors such as the type of vaccine and route of injection including intradermal, intramuscular, or subcutaneous are associated with the immune responses.^8^ Most Asian countries with a high rabies burden produced or imported nerve-tissue vaccines to provide PEP in public hospitals. Therefore, the introduction of intradermal vaccination was necessary due to its cost-effectiveness.^9^ In recent years, according to the WHO recommendation, the intradermal vaccination has been used for PEP.

 Post-exposure prophylaxis (PEP) against rabies includes wound washing, administration of rabies immunoglobulin, if necessary, and immediate vaccination. Performing these measures in the first hours after the animal bite can prevent the disease and subsequent death.^10^ Although the cost-effectiveness of PEP in the bitten cases has been proven,^11^ its effect depends on the timely initiation of PEP.^12^

 The increasing number of stray dogs and the increasing rate of animal bite cases in the country justify the need to pay more attention to the control of the disease and to carry out research on its various aspects.^13^ Analyzing the data available in the health system can be effective in increasing our knowledge of the epidemiologic aspects of rabies and removing the obstacles to the referral of bitten people. On the other hand, considering the huge costs imposed on the health system in order to provide free of charge rabies vaccine and human rabies immune globulin, it is necessary to ensure its effectiveness. Given that one of the factors affecting its effectiveness is timely vaccination, this study was designed to investigate the epidemiologic aspects of the animal bite and determine the factors related to the delay in initiation of PEP against rabies at the national level.

## Methods

 The current registry-based cross-sectional study was carried out on the bitten patients referred to the rabies treatment centers located in the health centers of Iran for receiving PEP against rabies from March 2021 to March 2022.

 In the rabies treatment center, demographic characteristics of the patient, as well as information related to the time and place of the bite, the type of biting animal, the characteristics of the wound, and the outcome of the exposure are recorded in the registry file of patients and also in the portal for the registration of bitten cases by the animal of Center for Disease Control and Prevention (CDC). In Iran, nearly 750 health centers in 31 provinces of the country are involved in PEP against rabies.^14^

 In this study, we obtained the data from CDC after obtaining ethical approval from the Ethics Committee of Hamadan University of Medical Sciences (IR.UMSHA.REC.1401.948). In this study, a delay in initiation of PEP was defined as the initiation of PEP against rabies more than 48 hours after a possible exposure to the rabies virus. According to the WHO, rabies exposure is divided into 3 categories as follows: category I: touching or feeding animals, as well as animal licks on intact skin, category II: nibbling of uncovered skin, minor scratches, or abrasions without bleeding, and category III: single or multiple transdermal bites or scratches, contamination of mucous membrane with the saliva of animals, injury in the head, face, neck, hands (fingertips to wrists), and genital area, type of bite by an animal with probable and confirmed rabies, exposure to bats, bitten or scratched by a bat, and people with weakness severe inherited and acquired immune system.^15^

 Data related to the Human Development Index (HDI) for each province was obtained from the “Planning and Budget Organization”. This index consists of four factors including mean years of schooling, expected years of schooling, life expectancy at birth, and gross national income per capita.

 The relationship between initiation of PEP against rabies and patients’ background, situation of the injury, and wound status were assessed using the chi-square test. The correlation between the rate of delay in initiation of PEP against rabies and HDI of the provinces was assessed using Pearson correlation test. Moreover, multivariate logistic regression was used to determine the predictors of delay in the initiation of PEP against rabies. The Hosmer-Lemeshow test was employed for model building and variables with a *P* value of less than 0.2 in the crude model were entered into the adjusted model. Data were analyzed using Stata software version 17.0. A *P* value of less than 0.05 was considered significant.

## Results

**Figure 1:**
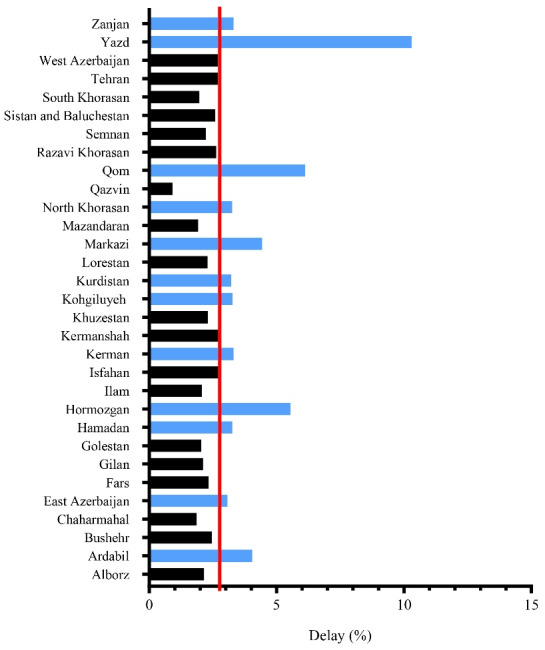
The rate of delay in initiation of PEP against rabies in different provinces (red line indicates national average (2.8%), provinces with blue color had a delay higher than national average and provinces with black color had a delay lower than national average)

 In the investigated time period, a total of 260 470 bitten patients referred for rabies PEP were investigated. The provinces of Golestan, North Khorasan, and Mazandaran had reported the highest incidence of animal bite (704.65, 631.9, and 557.86 per 100 000 population, respectively), and provinces of Bushehr, Kurdistan, and Hormozgan had the lowest incidence (131.08, 151.09, and 178.31 per 100 000 population, respectively). A total of 7285 cases (2.8%) had a delay of more than 48 hours in initiation of PEP. As shown in Figure 1, Yazd, Qom, and Hormozgan provinces had the highest rate of delay with the 10.3%, 6.12%, and 5.54% of the bitten cases and lowest rate of delay belonged to Qazvin, Chaharmahal and Bakhtiari, and Mazandaran with the 0.91%, 1.81%, and 1.92% of cases, respectively.

 Among the bitten cases, 201 164 (77.23%) were male and 254 611 cases (97.75%) were Iranian (5402 cases were Afghan, 325 were Iraqi, and 132 were from other countries). It was found that 56.39% of them lived in urban areas. A total of 11231 cases (4.31%) aged less than 5 years, and the mean age of the patients was 31.87 ± 18.52 years. In more than 98% of animal bite cases, the biting animal was domesticated and 37.86% of cases occurred in the afternoon (12-18 o’clock). There was a significant relationship between the initiation of PEP and patients’ gender, nationality, residence, animal type, season, and time of the event (*P* < 0.01) (Table 1).

**Table 1:** Initiation of PEP against rabies according to patient’s background and situation of the event

**Variables**	**Frequency**	**Percent**	**Vaccination time<48 hours**		**Vaccination time>48 hours**		***P *** **value**
			**Number**	**Percent**	**Number**	**Percent**	
Gender							0.001
Male	201164	77.23	196014	97.44	5150	2.56	
Female	59306	22.77	57171	96.40	2135	3.60	
Nationality							0.008
Iranian	254611	97.75	247523	97.22	7088	2.78	
Foreign	5859	2.25	5662	96.64	197	3.36	
Residence							0.001
Urban	146868	56.39	142810	97.27	4058	2.76	
Rural	90140	34.61	87429	96.99	2711	3.01	
Mobile	23462	9.01	22946	97.80	516	2.20	
Age group (year)							0.150
< 5	11231	4.31	10885	96.92	346	3.08	
5-15	45377	17.42	44128	97.25	1249	2.75	
16-30	67193	25.80	65332	97.23	1861	2.77	
31-45	72849	27.97	70868	97.28	1981	2.72	
46-60	39655	15.22	38516	97.13	1139	2.87	
+ 60	24165	9.28	23456	97.07	709	2.93	
Season of bite							0.001
Spring	70215	26.96	68437	97.47	1778	2.53	
Summer	64492	24.76	62741	97.28	1751	2.72	
Fall	63435	24.35	61575	97.07	1860	2.93	
Winter	62328	23.93	60432	96.96	1896	3.04	
Animal type							0.001
Domestic	256138	98.34	249031	97.23	7107	2.77	
Wild	1614	0.62	1534	95.04	80	4.96	
Redundant	2718	1.04	2620	96.39	98	3.61	
Time of bite (o’clock)							0.001
0-6	15874	6.09	15517	97.75	357	2.25	
6-12	80109	30.76	77670	96.96	2439	3.04	
12-18	98626	37.86	95792	97.13	2834	2.87	
18-24	65861	25.29	64206	97.49	1655	2.51	

 In Table 2, we compared the initiation of PEP against rabies based on wound status. As shown, in wounds with the perforation, rupture, or segregation of the tissue, the rate of delay in initiation of PEP was significantly low (1.69% and 1.42%, respectively, *P* < 0.001). Moreover, with the increase in the size and intensity of the wound, the rate of delay decreased (*P* = 0.001).

**Table 2:** Initiation of PEP against rabies according to wound status

**Wound status**	**Frequency**	**Percent**	**Vaccination time**				***P *** **value**
			**<48 hours**		**>48 hours**		
			**Number**	**Percent**	**Number**	**Percent**	
Bone fracture	66	0.03	64	96.97	2	3.03	0.910
Perforation	49,133	18.89	48021	97.62	1172	2.38	0.001
Rupture	6,634	2.55	6522	98.31	112	1.69	0.001
Segregation of the tissue	6,259	2.40	6170	98.58	89	1.42	0.001
Exposure category							0.001
I	5,381	2.07	5215	96.92	166	3.08	
II	175,381	67.33	170351	97.13	5030	2.87	
III	79,708	30.60	77619	97.38	2089	2.62	

**Figure 2:**
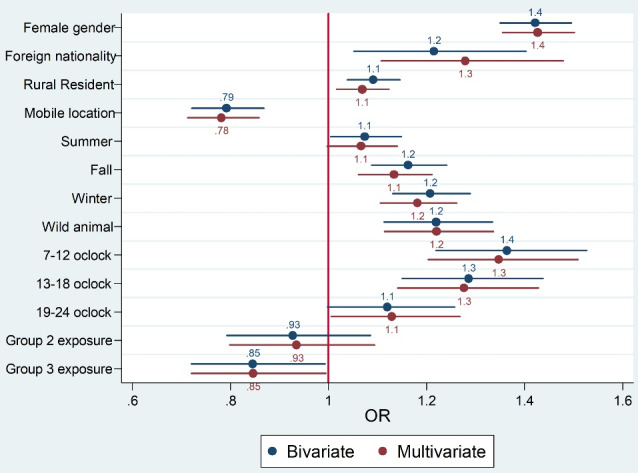
Predictors of delay in initiation of PEP against rabies (reference groups: male gender, Iranian nationality, urban area, spring season, domestic animal, time of exposure (0-6 o’clock), and exposure category I)

**Figure 3:**
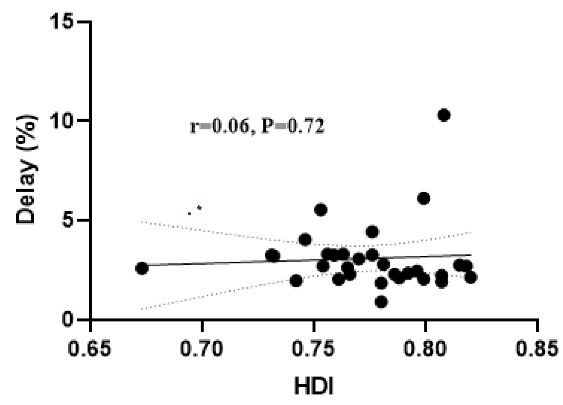
Correlation between the rate of delay in initiation of PEP against rabies and HDI of the provinces

 Results of the adjusted logistic regression showed that the odds of delay in initiation of PEP against rabies in females was 1.43 times higher compared to males (OR = 1.43, 95% CI: 1.36, 1.51, *P* < 0.001), in patients with a foreign nationality, it was 1.19 times higher compared to Iranian (OR = 1.19, 95% CI: 1.01, 1.12, *P* = 0.001), in rural residents, it was 1.07 times higher compared to urban residents (OR = 1.07, 95% CI: 1.01, 1.12, *P* = 0.010), in fall and winter seasons, it was 1.13 and 1.18 times higher compared to spring (*P* < 0.001), and in wild animals, it was 1.22 times higher compared to domestic animals (OR = 1.22, 95% CI: 1.12, 1.34, *P* < 0.001) (Figure 2). As shown in Figure 3, there was not a significant correlation between the rate of delay in the initiation of PEP against rabies and HDI of the provinces (r = 0.06, *P* = 0.720). In the mentioned time period, 15 deaths occurred due to the animal bite, 4 deaths were related to bleeding and infection and 11 deaths were due to rabies. In Table 3, the characteristics of the rabies cases were presented. No case of rabies occurred in the west of the country and Sistan and Balouchestan province, with the 3 cases of rabies being the highest reported rate. Additionally, 10 cases (90.09%) were male, 4 cases (36.36%) were self-employed, 4 (36.36%) were in the age group of 30-45 years, and 5 cases (45.45%) were urban residents. Besides, 6 cases (54.54%) of rabies occurred in the fall, and in 9 cases (81.81%), the biting animal was dog.

**Table 3:** The characteristics of the rabies cases

**Variables**	**Number **	**Percent**
Province		
Tehran	2	18.18
Alborz	1	9.09
Sistan and Balouchestan	3	27.27
Rezavi Khorasan	1	9.09
Mazandaran	2	18.18
Fars	2	18.18
Gender		
Male	10	90.90
Female	1	9.09
Job		
Self-employee	4	36.36
Housewife	1	9.09
Student	2	18.18
Child	1	9.09
Rancher	1	9.09
Worker	2	18.18
Residence		
Urban	5	45.45
Rural	5	45.45
Mobile	1	9.09
Age group (year)		
< 5	1	9.09
5-15	2	18.18
16-30	1	9.09
31-45	4	36.36
46-60	1	9.09
+ 60	2	18.18
Season of bite		
Spring	2	18.18
Summer	1	9.09
Fall	6	54.54
Winter	2	18.18
Animal type		
Dog	9	81.81
Fox	2	18.18

## Discussion

 The results of the present study showed that the rate of animal bite in Iran is approximately 334 per 100 000 population (Golestan with an incidence rate of 704.65 and Bushehr with an incidence rate of 131.08 per 100 000 population had the highest and lowest incidence rates, respectively), 2.8% of whom had a delay in initiation of PEP against rabies. The rate of delay is different among provinces and it does not have a relationship with the HDI of the communities. The rate of animal bites is higher in men and the reproductive age group of society (16-45 years). In spring, the number of bites is higher and bites by wild animals account for less than 1% of cases. Most bites occur in the afternoon (12-18 o’clock) and the rate of delay in initiation of PEP in females and residents of rural areas, bites with superficial injury, and bites occurring in winter and autumn is significantly higher. Additionally, in spite of the provision of PEP for the bitten people every year, 15 cases of rabies occurred in the country, which are not limited to a specific geographical area.

 The rate of delay in starting PEP was found to be different in different studies. In our study, a delay of more than 48 hours in PEP was observed in 2.8% of the cases, which is relatively satisfactory. Another study conducted in Isfahan showed that 30.8% of the children received PEP by more than 48 hours after exposure.^16^ In Ethiopia, a delay of more than 24 hours was observed in 47% of the cases and in two different studies conducted in India, a delay of more than 48 hours was reported in 59% and 9.04% of the cases.^17-20^ The difference in the level of access to rabies treatment centers and the level of awareness and attitude of people in different communities can justify these differences. Moreover, the difference in the definition of the delay is another reason. It is recommended that KAP (knowledge, attitude, practice) studies should be conducted in this regard to better understand the reasons for the delay in the initiation of PEP in different societies. The Persian version of the knowledge, attitude, and practice questionnaire for rabies (PKAP-Rabies) is now available.^21^

 Consistent with the results of the present study, results of two national studies conducted in Tabriz and Nahavand indicated higher rates of delay in the initiation of PEP in females.^22^ In the present study, the rate of delay in the initiation of PEP in rural residents was significantly higher. Similar results were shown in other studies conducted in Turkey and Tanzania.^23,24^ Difficulty in accessing public health services can also justify these discrepancies.

 In our study, we found that the rate of delay in PEP initiation in the winter and fall seasons is significantly higher. In another local study performed in Iran, a similar result was obtained.^22^ The reason for this can be attributed to the influence of weather conditions and difficulty in accessing rabies treatment centers. We found that only 2.07% of cases had category I bite and delay in PEP, which was significantly higher. In another study performed in India, consistent with our finding, only 2% of cases had category I bite.^18^ One reason is that patients with trivial injuries may not always seek medical care and underestimate the problem.

 Consistent with our finding, in some other studies,^18,25^ the rate of delay in PEP in patients with deep wounds was significantly lower. The reason for this finding is that patients with deep wounds visit health centers as soon as possible and refer to rabies treatment centers.

 In our study, the rate of delay in the initiation of PEP in foreign national cases was higher. It should be noted that the majority of them were refugees or illegal immigrants from Afghanistan (92.2%) residing in Iran. Results of the meta-analysis study investigating the most prevalent infectious diseases in Afghan refugees in Iran showed that a high proportion of them had tuberculosis, malaria, Crimean-Congo hemorrhagic fever, cholera, leishmaniasis, and hepatitis B.^26^ Therefore, these vulnerable populations need more attention.

 In our study, dogs are responsible for more than 80% of bitten cases and 9 out of 11 rabies cases were bitten by dogs. Similar findings were obtained from other studies.^13,27,28^ WHO estimates show that over 90% of the reported cases of rabies were bitten by dogs.^29^ Although vaccination can be effective in preventing human rabies, mass vaccination of dogs in developing countries is very challenging.^30^

 However, the present study had some limitations. First, the present study was registry-based and we could not have access to the data regarding some predictors such as educational level, the distance between their location and anti-rabies centers, and socio-economic class of animal bite victims. Second, in this study, only the referral cases of PEP have been examined; therefore, the information about non-referral cases has not been included.

**Highlights**

- A total of 260470 animal bite cases were observed in a year in Iran.
- Approximately 3% of cases had a delay in post-exposure prophylaxis ( > 48 hours).
- Women, foreign individuals, people living in rural areas, and those who were bitten by wild animals exhibited a higher likelihood of delay in initiating post-exposure prophylaxis.
- During the fall and winter seasons, there was a greater likelihood of delay in post-exposure prophylaxis compared to spring.

## Conclusion

 The timely initiation of PEP in bitten people in Iran is satisfactory. Delay in the initiation of PEP in rural residents and in winter and fall seasons indicates the role of accessibility in initiation of PEP for the prevention of rabies. Controlling the circulation of the rabies virus in the dog population through collaring domestic dogs, sterilization of stray dogs, and vaccination of domestic dogs plays an important role in controlling rabies in Iran.

## Acknowledgements

 The authors would like to express their gratitude to the Vice-Chancellor of the Research and Technology of Hamadan University of Medical Sciences for financially supporting the project (IR.UMSHA.REC.1401.948, Research code: 1401120210362).

## Authors’ Contribution

**Conceptualization:** Salman Khazaei, Erfan Ayubi.

**Data curation: **Jamshid Pourmozafari, Behzad Amiri, Mohammad Reza Shirzadi.

**Formal analysis:** Salman Khazaei, Erfan Ayubi.

**Funding acquisition: **Salman Khazaei.

**Investigation: **Salman Khazaei, Erfan Ayubi.

**Methodology:** Salman Khazaei, Behzad Amiri, Mohammad Reza Shirzadi.

**Project administration:** Salman Khazaei.

**Resources:**Salman Khazaei.

**Software: **Salman Khazaei, Erfan Ayubi.

**Supervision: **Mohammad Reza Shirzadi, Behzad Amiri.

**Validation: **Mohammad Reza Shirzadi.

**Visualization:** Mohammad Reza Shirzadi, Behzad Amiri.

**Writing–original draft:** Salman Khazaei, Erfan Ayubi.

**Writing–review & editing: **Salman Khazaei, Erfan Ayubi, Behzad Amiri.

## Competing Interests

 The authors declare that they have no conflict of interests.

## Funding

 This work was supported and funded by Hamadan University of Medical Sciences.
